# Daily living activities’ performance by male diabetics with sexual dysfunction in South Africa

**DOI:** 10.4102/phcfm.v15i1.3885

**Published:** 2023-07-10

**Authors:** Mabitsela H. Mphasha, Tebogo M. Mothiba, Linda Skaal

**Affiliations:** 1 Department of Public Health, Faculty of Healthcare Sciences, University of Limpopo, Polokwane, South Africa; 2 Dean’s Office, Faculty of Healthcare Sciences, University of Limpopo, Polokwane South Africa

**Keywords:** men living with diabetes, sexual dysfunction, coping with stress, fear of losing wife, activities of daily living

## Abstract

**Background:**

Sexual dysfunction (SD) is a common complication among men living with diabetes (MLWD), which adds to stresses induced by medical condition. Effect of stress on their daily living activities has been only poorly described.

**Aim:**

This study aimed to explore the behaviour and challenges of MLWD experiencing SD in respect of daily living activities.

**Setting:**

Five clinics in Senwabarwana in Limpopo province.

**Methods:**

Qualitative approach and phenomenological exploratory design were adopted to collect data from 15 male participants selected from five clinics using purposive homogeneous sampling. One-on-one interviews were conducted using voice recorders, and field notes were taken of non-verbal cues. Unstructured interview guide with principal question enabled instructive probing to be conducted. Data were analysed using eight steps of Tesch’s inductive, descriptive and open coding technique.

**Results:**

Participants reported stressful experiences, difficulty coping with diabetes and its accompanying complication of SD that led to fear of losing their wives. They indicated that as a result of stress and difficulty in coping with the condition, they were engaged in less physical activity than before their diagnosis.

**Conclusion:**

Sexual dysfunction is prevalent among male diabetics and often feel stressed and worried about losing their wives. They struggle to cope with conditions to the point where they are less capable of performing tasks than they were before diagnosis. These outcomes are critical issues that should be addressed in any diabetes treatment strategy.

**Contribution:**

Support-based collaboration of healthcare providers with spouses and revision of South African diabetes management strategy to incorporate healthy coping strategies are recommended.

## Introduction

A total of 537 million men and women aged 20–79 years are reported to be diagnosed with diabetes^1^ and even greater numbers with the undiagnosed condition. Furthermore, about 6.7 million people, which is equivalent to one every 5 seconds, died from diabetes in 2021.^1^ Male gender is considered to be a greater risk factor for the development of diabetes than female and hence the higher prevalence of diabetes among men, for reasons still not yet known.^2,3^ Although a Swedish study reported that the higher prevalence of diabetes in men was associated with their greater amount of visceral fat.^4^ The literature has also reported that diabetes has a marked effect on the sex life of diabetic men. High blood glucose levels, a symptom of diabetes, damage nerves and blood vessels essential for good sexual function, resulting in impaired sexual performance.^5^

Sexual dysfunction (SD) is a serious complication of diabetes mellitus^6^ and is common among men living with diabetes (MLWD). This condition is characterised by persistent sexual problems related to the man’s inability to attain and sustain an erection adequate for satisfactory sexual intercourse and pleasure.^7^ Sexual dysfunction, generally, is classified into four categories: (1) desire disorders, which are characterised by a lack of interest in sex; (2) arousal disorders, which are marked by inability to become physically stimulated or excited during sexual activity; (3) orgasm disorders, which are marked by a delay or absence of reaching climax and (4) experiencing pain, characterised by hurtful intercourse.^8^ In this context, SD implies any or combinations of a lack of sexual desire, inability to become physically aroused and to achieve and maintain satisfactory sexual engagement or early ejaculation. The global prevalence of SD ranges from 35% to 70% among diabetic men^9^ and is expected to affect 322 million males in the year 2025.^10^ As the prevalence of diabetes increases with age,^11^ so too does that of SD in men. Therefore, diabetes is a growing public health problem worldwide and indeed is the seventh leading cause of death in South Africa.^12^ The prevalence of SD among African men with diabetes mellitus (DM) is estimated at 72%, and in South Africa, it is reported to be 77%.^11^ It has been found that SD is two to three times more common in MLWD than in non-diabetic men.^5,13^ However, SD can exist before the diabetes is diagnosed, considering that millions of people have the undiagnosed condition.^14^ The screening of diabetes among men is therefore essential. Equally, awareness campaigns to address SD should be taken seriously.

Sexual health is a major component of human well-being. Sexual fulfilment and satisfaction are positively correlated with quality of life (QoL).^15^ Thus, having an impaired sex life and not feeling fulfilled sexually can lead to a lower QoL.^16^ Sexual activity is a type of bonding that can offer interested parties with a number of health benefits, as it promotes emotional intimacy, a more positive self-image for each partner, and stress reduction. The couple’s emotional intimacy increases partners’ adaptability and well-being and also improves quality of their relationship.^17^ Intimacy is regarded as the closeness, connectedness and bondedness felt in a committed partnership.^18^ Sex is a vital component of a person’s everyday life,^19^ and as such, it could affect activities of daily living (ADL), which are defined as fundamental skills required to manage basic physical needs.^20^ They are indicators of personal functional status and include abilities such as bathing, eating and mobility.^21^ Inability or difficulties in performing ADL are common occurrences among middle-aged and older adults.^22^ Being unable to perform ADL is also associated with a poor QoL, and subsequently increased healthcare costs and mortality rates. It is not yet fully understood how living with diabetes and SD impact ADL. Hence, the motivation for the study reported here that explored experiences and the performance of ADL among diabetic men with SD.

The literature on the subject revealed that MLWD commonly experience SD, resulting in poor or a lack of sexual fulfilment, which ultimately affects all aspects of their daily lives and increased stress.^17,20^ The findings of this study therefore are critical for further expanding on this topic to assess further the experiences of diabetic men. More specifically, this study sought to explore performance of ADL among MLWD and suffering SD in rural areas of Limpopo province.

## Research methods and design

A qualitative method and phenomenological exploratory descriptive study design was applied, in which 15 participants (MLWD with SD) were interviewed individually, to explore their experiences and performance of daily living activities.

### Study setting

The study was conducted in the rural Blouberg municipality in Senwabarwana, Capricorn District, Limpopo province, South Africa. The municipality supports 22 clinics, two health centres, four mobile clinics and one hospital. The clinics are divided into four sections, with two clinics from each area being purposefully sampled; however, in area 1, three clinics were sampled because of the high number of clinics with diabetes patients. There were five clinics included in all. Most persons residing in the Senwabarwana area observe a Sepedi culture. The customs of the Sepedi people are distinctive and fascinating. The philosophy of humanity, or *botho*, which emphasises compassion or caring for one another, is among the most intriguing.^23^

### Sampling and participants

A total of 15 male diabetics experiencing SD were purposively sampled from selected clinics in the Blouberg municipality. Data saturation was reached at participant 12; however, additional three participants were sampled for confirmation and added no new information; hence data collection was ceased. The sample was dependent on data saturation having been reached. Only male diabetics who had lived with diabetes for six or more months and were older than 18 years were included, because they can share lived experiences reliably and give consent to being interviewed.

### Data collection instrument and procedure

The data were collected through unstructured interviews using voice recorders and field notes taken of non-verbal cues observed. The interviews were conducted in Sepedi, which is a dominant language in the area and translated into English by language translator before transcripts were submitted to independent coder for analysis. The principal question for the interview was: ‘*What are your experiences of living with diabetes and sexual dysfunction*’? An additional question asked was: ‘*Are your daily living activities impacted by living with diabetes and sexual dysfunction*’? Probing and clarity-seeking questions were asked to obtain in-depth understanding of the responses to each question. The researcher used bracketing and reflective remarks during interviews. Bracketing in research is used to keep researchers, personal experiences apart from the subject under study, whereas reflection can be used to be reflection on participants’ interesting remarks to get clear understanding or dig more.^24^ The researcher’s facilitated interviews including observing and jotting down field notes from non-verbal cues. The researcher remained neutral, empathic and impartial throughout data collection while listening attentively and carefully and asking probing questions.

### Measures of rigour

A measure of rigour was ensured through trustworthiness by researchers observing credibility, transferability, confirmability and dependability.

#### Credibility

Prolonged engagement was applied through the use of first author, that is, Mphasha was responsible for data collection, and worked as a dietitian at the only hospital in Blouberg, which provides service delivery through outreach to the clinics. The researcher is known in the area and also stayed in the field for an extended period to build trust and rapport with the participants. For triangulation, researchers used both interviews and observations in the collection of data, which complemented each other in gaining insights. Member checks was ensured and thorough follow-up interviews were conducted with participants for confirmation after data analysis.

#### Transferability

Data saturation was ensured through collection of data until saturation was reached, which was achieved after 12 individual interviews. An additional three were sampled to confirm the findings. A detailed description was carried out through outlining fully describing study setting, target population and methodology for other researchers to conduct similar study in different setting.

#### Confirmability

Peer review was ensured through the use of independent coder who also analysed data. The researcher independently coded and recoded data and also developed themes. This ultimately led to a consensus meeting of the researcher and the coder for confirmation of the findings. Neutrality was ensured through researcher remaining neutral and was not emotional throughout the data collection and prolonged engagements.

#### Dependability

The researchers adhered to the fully described methods during data collection. Reliance on data collection tools, independent coder and supervisors was carried out through relying on performed interview guide, voice recorders, supervisors and independent coder.

### Data analysis

All interviews were audio-taped and then transcribed. The researchers used the services of a language translator to turn interviews conducted in Sepedi into English before analysis. Subsequently, all researchers analysed the verbatim transcripts independently and further procured the services of an independent coder. All researchers and the coder met in a consensus meeting and agreed on themes and sub-themes based on the ones that emerged when independently analysing the transcripts of interviews. Participants’ direct quotations were captured to support findings. Data were analysed using the eight steps of Tesch’s open coding qualitative data analysis method by Creswell.^25^

### Ethical considerations

This study is part of bigger study approved by Turfloop Research and Ethics Committee (No. TREC/35/2019: PG) and permission to conduct this study was granted by Limpopo Department of Health (ref: LP 201903-007) to access patients in the clinics. This article focuses on exploring experiences and challenges of performing ADL among diabetic men with SD. All participants provided written informed consent. Participation was voluntary, and participants of their right to withdraw from the study at any stage without penalty. Privacy and confidentiality of the participants data were also maintained.

## Results

### Socio-demographic profile

Table 1 shows that nine participants were pensioners aged 60 years and over. Six and five participants had primary and secondary education, respectively. Eleven had lived with diabetics for 5 years and more.

**TABLE 1:** Socio-demographic profile of participants.

Participant no.	Age (years)	Education	Time living with diabetes	Economic status	Relationship status
1	60	Secondary	5+ years	Pensioner	Married
2	65	Secondary	5+ years	Pensioner	Married
3	63	Tertiary	10+ years	Pensioner	Married
4	64	Primary	5–6 years	Pensioner	Married
5	58	Primary	15 years	Unemployed	Married
6	47	Primary	2 years	Unemployed	Married
7	69	Primary	23 years	Pensioner	Married
8	73	Primary	5 years	Pensioner	Married
9	80	Primary	18 years	Pensioner	Married
10	84	None	Since 1990s	Pensioner	Married
11	56	Secondary	8 months	Unemployed	Married
12	54	Tertiary	5 years	Unemployed	Married
13	52	Secondary	3+ years	Unemployed	Married
14	61	Tertiary	14 years	Pensioner	Married
15	56	Secondary	4 years	Employed	Married

### Themes and sub-themes that emerged from data analysis

#### Theme 1: Experiences

Living with diabetes and SD bring a psychological burden and may impact diabetes management, overall well-being and QoL. Participants described experiences of living with diabetes, as evident in the following sub-themes:

**Sub-theme 1.1. Stress:** Diabetes diagnosis is accompanied by or induces stressful experiences relating to the condition and its management. These may worsen in the presence of diabetes-related complications such as SD. This sub-theme shows that living with diabetes and SD experiences stress, as evident in the following quotations:

‘I also developed hypertension and diabetes-associated sexual dysfunction. This stresses me and makes me have sleepless nights even though, as a man, I’m adhering to health advice from healthcare professionals.’ (Participant 1, 60 years old, pensioner)

‘I really don’t know how to survive with this disease, which does not have a cure. I don’t sleep because it has been a long time now without having good sex with my wife. I’m afraid this will finish me.’ (Participant 4, 64 years old, pensioner)

‘Since my wife and I haven’t had real sex in years, I’ve been having trouble sleeping. I worry about where my wife gets her sexual pleasure.’ (Participant 12; 54 years old, unemployed)

**Sub-theme 1.2. Fear of losing their wives:** Living with diabetes is usually associated with phobic experiences. In this study, participants indicated that they feared losing their wives because of their inability to satisfy them with good sex. Sex is an important factor in a relationship, which enhances bonding, particularly among married couples. Here are some related statements recorded:

‘I’m 10 years older than my wife; and still have appetite for sex; my wife too has an appetite for sex as she is still younger. I’m unable to give her that pleasure she needs and deserves. I’m likely to lose my wife I married for many years, if nothing changes.’ (Participant 4, 64 years old, pensioner)

‘Inability to have sex with my wife brought me a lot of stress. Though my wife and I are done with having kids, we were doing sex for fun. However, nowadays we cannot have fun and I’m afraid she may get someone to have fun with, which may lead to her divorcing me.’ (Participant 5, 58 years old, unemployed)

‘I might lose my wife to whoever she is getting sexual pleasure from, even though I’m not sure if she does. Sex is vital and she is younger. I still need sex as well. I love her and don’t want to lose her because build family with her. Anyway, if she can leave me, I will have no one since I’m no longer capable of sexually satisfying any woman.’ (Participant 12, 54 years old, unemployed)

**Sub-theme 1.3. Difficulty in coping with condition:** Ongoing and chronic stress is problematic and may worsen a stressful experience, which includes response to stress or how to cope with the condition. Participants in this study indicated the difficulty of living with diabetes and SD, because sex is important to them. The following quotations support this conclusion:

‘Sometimes I feel like I should just die and rest, because I no longer enjoy anything. Inability to sexually satisfy the woman you married is very overwhelmingly.’ (Participant 5, 58 years old, unemployed)

‘I’m overwhelmed; I really cannot do anything. I’m always asking myself questions as to where will I end. I see no value in living because I cannot satisfy my wife sexually. I feel I will be better off if I’m dead.’ (Participant 6, 47 years old, unemployed)

‘I enjoy nothing, and I feel like I’m a lesser man because of my inability to satisfy my wife sexually. This stress will kill me more than diabetes because every second of my life I ask myself questions like, why should I live if I cannot have fun with my wife through sex; I’m no longer productive at work, too.’ (Participant 15, 56 years old, employed)

#### Theme 2: Challenges

Participants expressed challenges of living with diabetes and SD, which includes inability to perform work usually before diagnosis and as a result engaged in less physical activity. This is evident in the following sub-theme:

**Sub -theme 2.1. Inability to perform work done before diagnosis:** Stress can impact the health of patients in many ways, including compromising their QoL. Participants in this study indicated that they are unable to perform certain work they did before being diagnosed with diabetes and experiencing SD. This is evident in the following quotations:

‘I’m always weak; I no longer walk the longer distances I used to. I can’t perform heavy duties or even work for a prolonged time without resting many times.’ (Participant 5, 58 years old, unemployed)

‘I am unable to do hard labour because of body weakness and dizziness, which I used to do before diagnosis. I used to work at the farms doing hard labour before diagnosis.’ (Participant 6, 47 years old, unemployed)

‘I am no longer working like before I was diagnosis, in fact I just wakeup eat, sleep again or sit under a shade.’ (Participant 8, 73 years old, pensioner)

## Discussion

The aim of this study was to record the experiences and performance of ADL among men, living with diabetes and reporting SD, in rural areas of Limpopo. The authors learned that their study subjects are stressed, fear losing their wives and had difficulty coping with their conditions. Moreover, the study sample indicated that they are engaged in less physical activity because of their inability to perform duties carried out before their diagnosis as diabetics.

Participants reported experiencing stress brought on by diabetes and SD. The findings are consistent with a qualitative study conducted in three other parts of sub-Saharan Africa namely, Cape Town, Johannesburg and Lilongwe – which highlighted that sexual problems are serious complications of diabetes and are often accompanied by psychological issues.^26^ According to Kubler-Ross,^27^ the impact of diabetes diagnosis and its complications, particularly sexual problems, resemble stages of mourning, which are denial, anger, bargaining, depression and acceptance. A Brazilian qualitative study reported that the impact of diabetes is met with a mixture of feelings, which include preoccupation, panic and even anger and consequently denial of the condition.^28^ However, this study did not assess whether participants accepted the diagnosis of diabetes and its sexual impairment, but the stress the participants experienced. Feelings of stress are common among patients living with diabetes.^29^ Stress among diabetic patients was found to range between 12% and 23% prevalence rate.^29,30^ A diagnosis of diabetes brings its own stress, which is exacerbated by the need to change lifestyle and performance of ADL. The presence of stress among diabetics interferes with their ability to manage self-care activities and can affect blood glucose levels.^31^ Glycaemic control may be indirectly impaired by stress through its effects on the neuroendocrine system, because of changes in health-related behaviour.^32^ Stressful experiences of diabetes may also be associated with other complications.^33^ For example, chronic stress is an important risk factor for depression and depressed persons with diabetes have considerably lower QoL.^34^ Hence, it is desirable to conduct a study to assess the QoL of participants in order to design an appropriate intervention.

Our study population indicated that they have sleepless nights because of fear of losing their wives to other men as a consequence of their inability to engage in sexual activities. Moreover, diabetics are exposed to various other fears related to diagnosis, treatment, expected impact, prognosis and daily management of the condition.^35,36^ Normal sexual life is an important part of relationships; at the same time, the experience of sexual problems may affect self-image and self-esteem^28^ and the sense of masculinity of MLWD.

The study’s interviewees expressed difficulty coping with their diabetes diagnoses and sexual issues. Despite the fact that family is supportive, it became clear through probing that there is still a lack of coping among males with diabetes. Coping is defined as a psychological process developed at a conscious level and used to manage difficult and stressful situations in life.^37^ Healthy coping leads to physical adaptation, which is associated with improved QoL and adherence to treatment and better outcomes in the case of diabetes.^38^ Conversely, a lack of coping can lead to greater denial, non-adherence to treatment, depression and psychological distress.^39^ The American Association of Diabetes Educators^40^ has incorporated healthy coping within its strategic seven self-care behaviours to provide an evidence-based framework for the assessment, intervention and outcome (evaluation) measurement of the diabetic patient, programme and population. These seven behaviours are healthy eating, physical activity, taking medication, glucose monitoring, problem solving, healthy coping and reducing risks.^40^ So far, South Africa department of health has not yet incorporated coping within its strategy of diabetes care. This study has identified stress and difficulty in coping by diabetic men with impaired sexual function as a critical matter, requiring the attention of South African policymakers as a basis for comprehensive primary healthcare. Therefore, the South African department of health should adopt and incorporate coping or mental health measures in its advocacy of primary healthcare treatment for diabetes. Participants in this study reported that they helped them to cope, although it was not sufficient; hence it is recommended to further explore coping strategies among these male diabetics. Primary health care facilities should be facilitated with psychologists or social workers on a full-time basis to help patients manage and cope better with these conditions.

The interviewees in this study reported that they are engaged in less physical activity than previously because of the disruption of ADL. It was also found that the prevalence of stress among diabetics is linked with deterioration in their QoL and functionality, as reported elsewhere.^29,41^ Functionality in this context implies disruption of the performance of daily living activities or inability to perform work that used to be conducted before diagnosis. The World Health Organization^42^ regards QoL as an ‘estimation of well-being and measurement of health and the effects of health care’. However, a limitation of this study is that it did not assess the QoL of participants. Physical activity is crucial in the treatment of diabetes, improving QoL and overcoming complications such as SD.^14^ In addition, physical activity is associated with improved general well-being and reduction of stress.^43^ A physical activity assessment survey should therefore also be conducted in future surveys of this kind.

Most care of MLWD happens at home^44^ therefore the collaborative support of healthcare facilities and the spouses of participants are recommended in the case of the men who were interviewed. Family-centred care is crucial in the case of diabetics and helps in their stress management and ability to cope.^45^ This study did not establish obstacles to coping; therefore, it is important that a future study of this kind be conducted to understand these obstacles better. Furthermore, an appropriate physical activity programme for diabetics should be designed with the involvement of their family members. The authors also recommend the establishment or strengthening of support groups for MLWD to assist them overcome their experiences of stress with the help of healthcare providers. It is African culture of Bapedi to care for one another, which would make it easier to establish family support system for patients.

### Limitations

The findings of this study are based on a relatively small sample of 15 males living with diabetes in Senwabarwana in Limpopo province. However, the authors believe that they are broadly applicable to other populations in similar circumstances elsewhere.

## Conclusion

This study establishes that MLWD and experiencing SD are stressed because of their state of health and inability to satisfy their partners sexually. Moreover, MLWD suffering SD fear losing their wives to other men and have difficulty in coping with these circumstances. Hence, the authors consider stress to be a critical issue among diabetic men. It is recommended that the South African department of health revise its strategy for the treatment of diabetes, and incorporate healthy coping methods. The coping methods should be benchmarked, adapted, and adopted as a key element in its management. Conversely, a study regarding obstacles to healthy coping should be conducted. The support-based collaboration of healthcare providers with spouses of MLWD and experiencing SD is encouraged to ameliorate their condition.
